# Decolonizing Global Health Research: Perspectives from US and International Global Health Trainees

**DOI:** 10.5334/aogh.3961

**Published:** 2023-02-06

**Authors:** Matthew DeCamp, Limbanazo Matandika, Lameck Chinula, Jorge L. Cañari-Casaño, C. Hunter Davis, Emily Anderson, Marlena McClellan, Benjamin H. Chi, Valerie A. Paz-Soldan

**Affiliations:** 1 Division of General Internal Medicine and Center for Bioethics and Humanities, University of Colorado Anschutz Medical Campus, US; 2 Center for Bioethics in Eastern and Southern Africa, College of Medicine, University of Malawi, MW; 3 University of North Carolina – Project Malawi and Department of Obstetrics and Gynecology, University of North Carolina at Chapel Hill, US; 4 School of Public Health and Administration, Universidad Peruana Cayetano Heredia, PE; 5 Institute for Global Health and Infectious Diseases, University of North Carolina at Chapel Hill (current affiliation: Center on Gender Equity and Health, University of San Diego California), US; 6 Department of Obstetrics and Gynecology, University of North Carolina at Chapel Hill, US; 7 Tropical Medicine, Tulane School of Public Health and Tropical Medicine, US

**Keywords:** Decolonizing global health, global health education, global health ethics

## Abstract

**Background:**

“Decolonizing global health” (DGH) may help global health trainees understand and remediate the effects of historical colonialism on global health, but little is known regarding how trainees perceive DGH. Understanding their perspectives is critical for designing educational interventions tailored to their needs.

**Objectives:**

To understand how trainees perceive DGH research and to determine if perspectives differ between trainees from high- (HICs) versus low- and middle-income countries (LMICs).

**Methods:**

An online survey of all 2017–2022 pre-doctoral and post-doctoral trainees (n = 111) and mentors (n = 91) within a multi-university program that supports US and international investigators in one-year mentored global health research. The survey evaluated individuals’ self-reported knowledge and attitudes toward DGH and their perceptions of historical colonialism’s impact on eight aspects of global health.

**Findings:**

The response rate to trainee surveys was 56%. Trainees from LMICs were less aware of the concept of DGH; 5/25 (20%) had never heard of DGH and 16/25 (64%) reported that they “know a little,” whereas all HIC trainees had heard of DGH and 29/36 (81%) reported that they “know a little” (p = 0.019). For three aspects of global health (i.e., which research questions get asked; development of collaborative relationships; and data/statistical analyses), trainees from LMICs were more likely to report positive effects of colonialism. However, in open-ended responses, no thematic differences existed between how LMIC and HIC trainees defined DGH (i.e., actively eliminating power imbalances; prioritizing local needs; promoting local leadership; providing equitable opportunities; and ensuring programs are culturally appropriate).

**Conclusions:**

Different perspectives surrounding what DGH means suggest a shared understanding may be needed and is arguably prerequisite to designing educational interventions to help global health trainees recognize, understand, and act in global health. Future research is needed to understand perspectives on decolonization across diverse contexts with attention to constructs such as race, ethnicity, and gender.

## Background

Global health has long wrestled with a past characterized by both humanitarianism and shameful subjugation, disrespect, and harms. The legacy of medical missionaries looms large over global health. Missionaries sometimes disrespected local cultural beliefs or, at times, were entangled in (or used for) political motivations. More recent examples of global health’s mixed past include colonial influences and inequities within global health funding institutions, organizations, and partnerships [1,2,3]. This has manifested in issues such as who sets funding priorities and agendas, whose voices are heard, and even which countries’ needs are prioritized (e.g., regarding COVID-19 vaccines). Coming to terms with this checkered past, built upon tropical medicine, hygiene, and international health, requires ongoing reflection and action for those in global health. For some the very concept of global health reflects colonialism, privileging a Western mindset [4].

Reflecting this, in the past several years, there has been increasing attention to the concept of “decolonizing” global health. The concept of decolonization is not new. Its historical and conceptual roots lie in the ideas of ending both colonial rule (i.e., political decolonization) and neocolonialism (i.e., removing those financial, social, and cultural influences that maintain control and restrict self-determination) [5]. Neither is it entirely new in global health to apply concepts with similarities to decolonization. In global efforts to control the HIV epidemic, for example, decades of efforts by civil society and advocacy groups have long sought to eliminate inequities in power and control [6]. Even if not described as decolonizing movements *per se*, one can see parallels. The need for reducing disparities and inequities in health and access at local and global scales is relevant to the concept of decolonization. For example, this need directs us to reflect on who holds power in funding allocation, for prioritizing research topics, and in building research capacity [7,8].

Nevertheless, the concept of decolonizing global health appears important, now more than ever. The COVID-19 pandemic has again revealed colonial and neocolonial influences in global health [9]. Despite decades of efforts to make health care more equitable globally, at the time of COVID-19 vaccine distribution at the global scale, the world failed, suggesting a need to rethink global health knowledge, leadership, policy, and thought [2]. This includes rethinking global health research training [10]. The historical export of Euro-American medical schools and research partnerships have been criticized for maintaining a colonial-like focus of power and control in high-income countries (HICs) [11]. Frameworks now exist for how to structure global health partnerships that acknowledge medicine’s past role in colonialism and the need to address power dynamics and equalize learning opportunities in global health [12,13].

There is widespread recognition of the need to educate global health trainees about the effects of colonialism on global health today. However, little is known regarding how global health trainees understand what decolonization of global health research means. Understanding their perspectives is critical for the design of educational interventions tailored to their needs. In addition, there is little evidence to suggest that all involved in global health share a common understanding of decolonizing global health research, or what decolonization practically requires. The concept and its implementation have been critiqued for inadvertently reinforcing colonialism, or for being insufficient to real-world problem solving [14,15].

In this study, we sought to understand the perspectives of global health trainees related to the concept of decolonizing global health research. This included trainees’ self-reported knowledge and attitudes toward the concept as well as the perceived effect of colonialism on global health funding, priorities, career advancement, and other aspects of global health. We also sought to determine if these perspectives differ between trainees from HICs and those from LMICs.

## Methods

### Study setting

This study was conducted as part of the Global Health Fellows and Scholars Program, funded by the Fogarty International Center and other participating institutes and centers at the US National Institutes of Health. Specifically, we engaged trainees from the UJMT Consortium, comprising principal and collaborating faculty from the University of North Carolina at Chapel Hill, Johns Hopkins University, Morehouse School of Medicine, and Tulane University. The UJMT Consortium has been funded to support global health research training efforts since 2012 and has provided training opportunities to more than 200 trainees (described in greater detail elsewhere [16]). At the time this study was conducted, the UJMT Consortium was one of six such groups that supported US and international trainees in one-year mentored global health research attachments.

We engaged trainees from the prior five years of the program (2017–2022), including pre-doctoral and pre-professional trainees from HICs (i.e., US citizens or permanent residents) and postdoctoral and post-professional trainees from both the U.S. and LMICs. Training sites were located in Bangladesh, Democratic Republic of the Congo, Ghana, India, Malawi, Mexico, Peru, Sierra Leone, South Africa, Suriname, Uganda, Vietnam and Zambia. From 2017–2022, the top four training sites accounted for 76/111 (68%) of trainees (29 in Malawi; 19 in Peru; 16 in Uganda, and 12 in Zambia). All trainees in the UJMT Consortium have English language proficiency.

### Sample

We created our sample from the complete list of trainees and mentors within the most recent five years of the UJMT program (2017–2022). We chose the most recent five years because attention to decolonizing global health is a relatively recent phenomenon and because surveying recent trainees was better suited to the broader projects’ goal of developing new ethics and decolonizing global health education materials (e.g., to reduce recall bias in surveying trainees from 10 years prior). All 111 trainees from this time period were sent emails asking them to participate. We also sent recruitment emails to 91 active mentors from the UJMT Consortium who have supported at least one trainee in the program, asking them to complete a survey.

### Survey instrument

After reviewing the literature, we found no validated items related to the concept of decolonizing global health; therefore, we created two *de novo* survey instruments (one geared toward global health trainees; another, toward global health mentors) based on current literature and the research team’s experience. The goals of the survey were to understand individuals’ self-reported knowledge and attitudes toward decolonizing global health and to evaluate perceptions of the impact of historical colonialism on eight aspects of global health today: (1) funding available for global health; (2) research questions in global health; (3) research questions that get prioritized for funding; (4) developing collaborative relationships in global health; (5) data/statistical analyses used in global health; (6) authorship decisions on global health; (7) equal career advancement opportunities; and (8) global health research, overall.

The survey went through multiple rounds of feedback and revision to ensure face and content validity. First, it was reviewed by a Technical Advisory Panel comprised of nine individuals from diverse geographic locales and with diverse expertise in global health and global health education. After revision, we conducted five cognitive interviews with three global health trainees and two global health mentors, who represented the intended audience for our survey. Following iterative refinement, the final instrument included 36 core items and required 15–20 minutes to administer (see Appendix).

### Survey administration

The survey was conducted online from February 2022 to April 2022 via Qualtrics (Provo, UT). It was optimized for both desktop and mobile format. Survey respondents were given the option of being entered into a random chance drawing to win a tablet computer device. We sent several email reminders by email to encourage participation.

### Data processing and analysis

For analysis of quantitative data, responses were exported for analysis in Stata 17.0 (Stata Corporation, College Station, Texas, USA), which is a secure, password-protected software platform. Only fully completed surveys were included in the final analysis. One survey was only 40% complete and was removed from the sample. Another survey was missing only the self-identified gender item and was included.

Some variables were combined to aid in interpretation and to improve statistical power. For example, we assessed aspects of global health on a 7-point Likert scale from –3 (very negative) to +3 (very positive). Recognizing that this scale was not proven valid to differentiate, e.g., –3 versus –2, these data were analyzed by combining responses of –3, –2, and –1 into one category (“Negative”) and by combining +1, +2, and +3 into one category (“Positive”). Similarly, we assessed participants’ attitudes and emotions toward the concept of decolonizing global health on a 5-point Likert scale; these data were analyzed by combining “Strongly Disagree” and “Disagree” into a single category and by combining “Strongly Agree” and “Agree” into a single category. Unless otherwise noted, data were analyzed using Pearson Chi-squared tests and Fisher Exact Tests (for bivariate comparisons with small n’s).

For analysis of qualitative data, recognizing the limitations of open-ended survey response, we employed basic content analysis. Three members of the team (MD, MM, and LM) reviewed responses to generate initial codes of thematic content. Together they refined and elucidated definitions of these codes. Then, all the responses were re-coded independently with any disagreements in coding resolved by discussion to arrive at a final set of coded responses. For each response, we identified one *primary* theme (i.e., we did not allow for more than one coded theme per response). Given our interest in comparing LMIC to HIC trainees, coders were blinded to whether a response came from an LMIC or HIC trainee, in order to promote unbiased analysis.

### Ethics approval

The study was reviewed and approved by Institutional Review Board at the University of North Carolina at Chapel Hill (IRB#21-2994). All participants gave their voluntary, informed consent to participate. The study was conducted in accord with the Declaration of Helsinki.

## Findings

Among the 111 trainees in our study period (2017–2022), we were unable to contact two individuals. Of those who received the request for participation, 61/109 (56%) successfully completed the survey. The response rate to the mentor survey was significantly lower than that of the trainees. Of the 91 mentors, two recruitment emails were returned as undeliverable. Of those who received the request for participation, 26/89 (29%) completed the survey. Given this low response rate among mentors, we present mentor data only where comparison to trainee data yielded insights for future research hypotheses.

### Characteristics of respondents

Characteristics of survey respondents are in Table 1. We noted that respondents from HICs were more likely to report female gender, a finding consistent with overall UJMT participation. Among all HIC trainees from 2017–2022, 55/69 (80%) reported female gender and 14/69 (20%) reported male gender. In comparison, over the same five years, 18/42 (43%) of LMIC trainees reported female gender and 24/42 (57%) LMIC trainees reported male gender. We observed that trainees from LMICs were more likely to report having no prior experiences in global health training (which we defined broadly to include both local and international programs, clinical and non-clinical). Trainees from LMICs were also more likely to report conducting implementation science research.

**Table 1:** Characteristics of Respondents (n = 61).


CHARACTERISTIC	ALL (N = 61)	HIC (N = 36)	LMIC (N = 25)	P VALUE

**Gender^±^**				

Female	35 (58.3%)^¥^	27 (75.0%)	8 (33.3%)	0.001^a^

Male	25 (41.7%)^¥^	9 (25.0%)	16 (66.7%)	

**Training Stage**				

Post-doctoral	45 (73.8%)	28 (77.8%)	17 (68.0%)	0.393^a^

Pre-doctoral	16 (26.2%)	8 (22.2%)	8 (32.0%)	

**Degree Status at Time of UJMT Program**				

Already had MD	28 (45.9%)	15 (41.7%)	13 (52.0%)	0.095^b^

Already had PhD	9 (14.6%)	4 (11.1%)	5 (20.0%)	

Pursuing MD	7 (11.5%)	7 (19.4%)	0 (0.0%)	

Pursuing PhD	17 (27.9%)	10 (27.8%)	7 (28.0%)	

**Area of Study at time of UJMT Program**				

Basic Science	12 (19.7%)	10 (27.8%)	2 (8.0%)	0.099^b^

Secondary Data Analysis	13 (21.3%)	9 (25.0%)	4 (16.0%)	0.399^a^

Clinical Trial	11 (18.0%)	8 (22.2%)	3 (12.0%)	0.500^b^

Public Health/Health Education	30 (49.2%)	19 (52.8%)	11 (44.0%)	0.500^a^

Implementation Science	11 (18.3%)	2 (5.6%)	9 (36.0%)	0.005^b^

**Prior Experiences in Global Health Training**				

Never	13 (21.3%)	2 (5.6%)	11 (44.0%)	<0.001^b^

1–2	26 (42.6%)	17 (47.2%)	9 (36.0%)	

3–5	14 (23.0%)	13 (36.1%)	1 (4.0%)	

More than 5	8 (13.1%)	4 (11.1%)	4 (16.0%)	


^±^ Total n in this row equals 60 – one participant did not answer about gender. ^a^ Chi-square test. ^b^ Fisher’s exact test.

We were able to compare respondents to non-respondents within the UJMT program, and found no differences between respondents and non-respondents in terms of gender, LMIC versus HIC status, or program year, but we did find that respondents were more likely to be post-doctoral (i.e., 45/61 [74%] compared to 25/50 (50%) non-respondents). (See Supplementary Material Table S1).

### Self-reported knowledge and attitudes toward decolonizing global health

Respondents’ self-reported knowledge and attitudes toward decolonizing global health are in Table 2. We found that 29/36 (81%) of respondents from HICs reported that they “know a little” about the concept of decolonizing global health, and all had heard of the concept. In contrast, participants from LMICs reported less overall knowledge of the concept; 5/25 (20%) had never heard of it and 16/25 (64%) reported that they “know a little.” This finding was statistically significant (p = 0.019).

**Table 2:** Participants’ Self-reported Knowledge and Attitudes Toward Decolonizing Global Health.


ITEM	ALL (N = 61)	HIC (N = 36)	LMIC (N = 25)	P VALUE

**Overall Knowledge of Decolonization of Global Health**				

Never heard of it	5 (8.2%)	0 (0.0%)	5 (20.0%)	0.019^b^

Know a little	45 (73.8%)	29 (80.6%)	16 (64.0%)	

Know a lot	11 (18.0%)	7 (19.4%)	4 (16.0%)	

**“I am supportive of the current movement toward decolonization of global health.”**				

Strongly Disagree/Disagree	0 (0.0%)	0 (0.0%)	0 (0.0%)	1.000^b^

Neutral	4 (6.6%)	2 (5.6%)	2 (8.0%)	

Agree/Strongly Agree	57 (93.4%)	34 (94.4%)	23 (92.0%)	

**“I am worried that the current movement toward decolonization of global health will do more harm than good.”**				

Strongly Disagree/Disagree	45 (73.8%)	26 (72.2%)	19 (76.0%)	0.274^b^

Neutral	9 (14.8%)	4 (11.1%)	5 (20.0%)	

Agree/Strongly Agree	7 (11.5%)	6 (16.7%)	1 (4.0%)	

**“I feel excited because decolonization will give more opportunities for people in LMICs to succeed in global health research.”**				

Strongly Disagree/Disagree	0 (0.0%)	0 (0.0%)	0 (0.0%)	0.430^b^

Neutral	7 (11.5%)	3 (8.3%)	4 (16.0%)	

Agree/Strongly Agree	54 (88.5%)	33 (91.7%)	21 (84.0%)	

**“I feel defensive like I am being accused of doing something wrong.”**				

Strongly Disagree/Disagree	46 (75.4%)	29 (80.6%)	17 (68.0%)	0.126^b^

Neutral	13 (21.3%)	5 (13.9%)	8 (32.0%)	

Agree/Strongly Agree	2 (3.3%)	2 (5.6%)	0 (0.0%)	

**“I feel hopeful that decolonization will actually reduce global health inequalities”**				

Strongly Disagree/Disagree	2 (3.3%)	2 (5.6%)	0 (0.0%)	0.106^b^

Neutral	14 (23.0%)	11 (30.6%)	3 (12.0%)	

Agree/Strongly Agree	45 (73.8%)	23 (63.9%)	22 (88.0%)	

**“I feel guilty because I personally benefitted from colonialism.”**				

Strongly Disagree/Disagree	29 (47.5%)	12 (33.3%)	17 (68.0%)	0.001^a^

Neutral	16 (26.2%)	9 (25.0%)	7 (28.0%)	

Agree/Strongly Agree	16 (26.2%)	15 (41.7%)	1 (4.0%)	


^a^ Chi-square test. ^b^ Fisher’s exact test.

In the set of questions related to respondents’ attitudes toward the concept, we found that, on the whole, most respondents from LMICs and HICs were supportive of the movement toward decolonization of global health (57/61, 93%), few were worried that it could do more harm than good (7/61, 12%), and most (54/61, 89%) were excited that decolonizing global health would give more opportunities for people in LMICs to succeed. There was relatively less support for the idea that decolonization would actually reduce global health inequalities, with 45/61 (74%) agreeing or strongly agreeing and 14/61 (23%) neutral on this topic.

The only statistically significant difference between HIC and LMIC respondents related to feelings of guilt. Respondents from HICs (15/36, 42%) were more likely to agree or strongly agree with the statement that they “feel guilty because I personally benefitted from colonialism” whereas only a single individual from a LMIC agreed with this statement and 17/25 (68%) disagreed or strongly disagreed (p = 0.001).

When examining the 26 total mentor surveys, this statement around feelings of guilt was an area where we noted a possible difference when compared to the trainee surveys. We observed a potential difference in the attitudes of HIC mentors. Compared to the 42% of HIC trainees who agreed or strongly agreed regarding guilt, only 2/16 (13%) of HIC mentors did so, and 9/16 (56%) disagreed or strongly disagreed with the statement that they felt guilty for having benefitted from colonialism.

### Themes in how trainees define decolonizing global health

Our survey asked respondents to give a 1-sentence summary of what decolonizing global health means to them. Eight individuals responded, “I don’t know,” leaving 53 open-ended responses for analysis. Summary results of our qualitative content analysis are in Table 3.

**Table 3:** Main categories of responses in defining what decolonizing means.


	LMIC RESPONDENTS (N = 20)	HIC RESPONDENTS (N = 33)	TOTAL

**Actively eliminating power imbalances**	7 (35%)	14 (42%)	21

**Prioritizing local needs**	4 (20%)	7 (21%)	11

**Promoting local leadership**	5 (25%)	5 (15%)	10

**Providing equitable opportunities for all**	3 (15%)	6 (18%)	9

**Ensuring programs are culturally appropriate**	1 (5%)	1 (3%)	2


We found that responses fitted into five general categories, with no clear difference in responses based on whether a trainee was from an LMIC or HIC. The most common responses related to active elimination of historical and colonial power imbalances. These responses included words and phrases such as the following: “intentional undoing,” “fights against the system of dominance,” “critique imperialism,” “dismantling colonial legacies,” or “removing the superiority of one population.”

The second most common category of responses focused on increasing the prioritization of local needs, without necessarily referencing the elimination of existing institutional structures. These responses included phrases such as, “re-centering global health priorities around local needs and interests,” or “reprioritizing global health studies to focus on the goals, values, and question in the population of study.”

The third most common theme related to an emphasis of local leadership and decision-making, again separate from the active elimination of power structures (and recognizing that prioritizing local needs may or may not imply local leadership). Here, responses emphasized “empowering developing countries to take lead on such collaborations” or “making global health a concept that the ‘global south’ actively participates in and influences.”

### Perceptions of the impact of colonialism on global health

In Table 4, we present respondents’ perceptions of the impact of historical colonialism on global health today. In general, trainees from LMICs had mixed perceptions whereas trainees from HICs had generally negative perceptions of this impact. To illustrate, when asked about the impact of colonialism on which questions get asked in global health, 12/25 (48%) of LMIC respondents assessed this as positive compared to 5/36 (14%) of HIC respondents. However, 11/25 (44%) of LMIC respondents had a negative assessment. The differences in the overall distribution were statistically significant (p = 0.012). We observed a similar finding regarding the development of collaborative relationships in global health, where 16/25 (64%) LMIC respondents assessed this as positive and 25/36 or 69% of HIC respondents assessed this as negative (p < 0.001). Finally, we included an item intentionally chosen to be potentially seen as more neutral, that is, the effect of colonialism on the data/statistical analyses used in global health research. We found that HIC respondents saw this as predominantly neutral (22/36, 61%) whereas LMIC respondents saw this as positive (12/25, 48%; p < 0.001).

**Table 4:** Perceptions of the Impact of Historical Colonialism on Aspects of Global Health.


*WHAT DO YOU THINK THE IMPACT OF COLONIALISM HAS BEEN ON…*	All (N = 61)	HIC (N = 36)	LMIC (N = 25)	P VALUE

**….the total amount of** **funding** **available for global health today**				

Negative	23 (37.7%)	12 (33.3%)	11 (44.0%)	0.602^a^

Neutral	13 (21.3%)	9 (25.0%)	4 (16.0%)	

Positive	25 (41.0%)	15 (41.7%)	10 (40.0%)	

**…which** **research questions** **get asked in global health.**				

Negative	35 (57.4%)	24 (66.7%)	11 (44.0%)	0.012^a^

Neutral	9 (14.8%)	7 (19.4%)	2 (8.0%)	

Positive	17 (27.9%)	5 (13.9%)	12 (48.0%)	

**…. which** **research questions get prioritized** **for funding in global health.**				

Negative	40 (65.6%)	27 (75.0%)	13 (52.0%)	0.088^a^

Neutral	3 (4.9%)	2 (5.6%)	1 (4.0%)	

Positive	18 (29.5%)	7 (19.4%)	11 (44.0%)	

**…developing** **collaborative relationships** **in global health research.**				

Negative	31 (50.8%)	25 (69.4%)	6 (24.0%)	<0.001^b^

Neutral	8 (13.1%)	5 (13.9%)	3 (12.0%)	

Positive	22 (36.1%)	6 (16.7%)	16 (64.0%)	

**…the** **data/statistical analyses** **used in global health research.**				

Negative	19 (31.2%)	11 (30.6%)	8 (32.0%)	<0.001^a^

Neutral	27 (44.3%)	22 (61.1%)	5 (20.0%)	

Positive	15 (24.6%)	3 (8.3%)	12 (48.0%)	

**…****authorship** **decisions on global health papers.**				

Negative	39 (63.9%)	27 (75.0%)	12 (48.0%)	0.092^b^

Neutral	11 (18.0%)	5 (13.9%)	6 (24.0%)	

Positive	11 (18.0%)	4 (11.1%)	7 (28.0%)	

**…****equal career advancement opportunities** **for all people in global health.**				

Negative	41 (67.2%)	28 (77.8%)	13 (52.0%)	0.060^b^

Neutral	5 (8.2%)	3 (8.3%)	2 (8.0%)	

Positive	15 (24.6%)	5 (13.9%)	10 (40.0%)	

**…** **global health research** **, overall.**				

Negative	35 (57.4%)	24 (66.7%)	11 (44.0%)	0.129^b^

Neutral	6 (9.8%)	4 (11.1%)	2 (8.0%)	

Positive	20 (32.8%)	8 (22.2%)	12 (48.0%)	


^a^ Chi-square test. ^b^ Fisher’s exact test.

**Figure 1:**
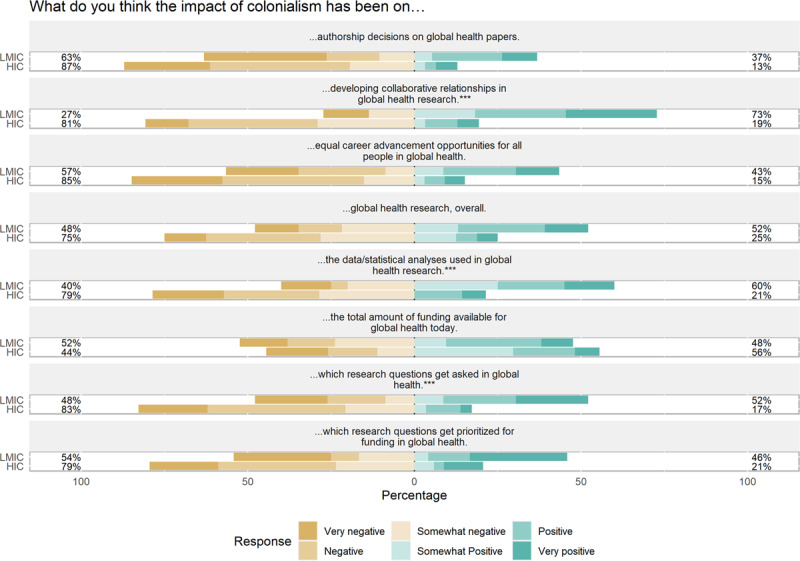
Perceptions of the Impact of Colonialism on Several Aspects of Global Health (*** indicates statistically significant findings).

In Figure 1 we present graphically the comparisons in Table 4 between LMIC and HIC respondents’ perceptions of the impact of historical colonialism on global health today. (See Supplemental Materials Figure S1 for aggregate data from all respondents.) For better visualization, we plot these perceptions by removing the “Neutral” category from the eight items. For all but one prompt (“the total amount of funding available for global health today”), LMIC respondents more frequently had a positive assessment in response to our questions. This allows the overall trend of the greater perceived negative impact of colonialism among HIC respondents compared to LMIC respondents to be seen more clearly.

Within the items on perceptions of the impact of colonialism on global health, we observed a potential difference in the mentor responses. While limited by a small sample (n = 26 responses; 16 HIC, 10 LMIC), we did not see evidence that LMIC mentors were generally more positive than HIC mentors regarding the effect of colonialism. Across all items, at least 6/10 (60%) of LMIC mentors perceived the impact of colonialism to have been negative. On four of the eight items, 8/10 (80%) of LMIC mentors expressed negative perceptions of the impact of colonialism on global health. See Supplemental Materials Table S2 for tabular presentation of these data.

Our relatively small sample on a 7-point scale limits our ability to do additional analyses. However, we conducted several analyses to help ensure the validity of these findings. For example, we compared results when including the neutral category in the “Negative” impact category and when including the neutral category in the “Positive” impact category; neither affected our results. We also analyzed using the mean values on the full –3 to +3 scale; all three findings remained statistically significant. Using the Mann-Whitney test, only the question about collaborative relationships remained statistically significant (See Supplementary Materials Table S3).

We attempted to analyze for interactions between site of LMIC/HIC status and gender. We found no consistent findings in the direction or magnitude of the effect size (e.g., on two items where the LMIC/HIC difference was statistically significant, no gender effect was observed). For example, when comparing females to males within the HIC and LMIC categories, the differences seen were not statistically significant. However, our analysis was limited by our small sample size (more than half of the cells had an n < 5), which was not sufficient to produce reliable estimates on the basis of gender (See Supplemental Materials Table S3).

## Discussion

To our knowledge, we have conducted one of the first empirical studies regarding global health trainees’ perspectives about the contemporary movement toward decolonizing global health within global research partnerships. Our study found differences in knowledge and attitudes between LMIC and HIC trainees that have important implications for global health research training.

First, our findings suggest the need for the global health community to develop clarity around exactly what decolonizing global health means. Recent studies involving global health leaders have revealed both agreement and disagreement about the precise meaning of decolonizing global health [17,18], and decolonizing global health is at present a contested concept [14]. Our respondents’ definitions of the concept and their attitudes toward it reflect the significant differences present in the literature around decolonizing global health. For instance, to some, decolonizing is about ending supremacy [13,19] and the dominance of HIC institutions in global health by actively eliminating that supremacy. For others, the need is less about breaking down HIC power and more about equalizing it or positively empowering LMIC individuals and institutions for the sake of equity [20]. To build reflection and action toward decolonizing global health, we must first be able to recognize and understand it.

Coming to terms with these different perspectives is critical for global health education. A common understanding of decolonizing global health could better facilitate the design and content of educational activities. What does the concept of decolonizing global health uniquely add to prior work around equity and research partnerships in global health, and what are its implications for education? Our findings suggest the opportunity and need for dialogue on this question. Decolonizing global health has rhetorical appeal, but there is a need to avoid superficial attention to the concept. Importantly, this dialogue should occur not only between HICs and LMICs; it must also occur within each setting (as the effect of colonialist attitudes are also evident within countries) [15] and between and within different types of stakeholders. Some have argued how the decolonizing movement suffers from an overemphasis on intercountry relationships [21].

Second, the differences in attitudes toward decolonization that we observed between LMIC and HIC research trainees require additional research. In general, LMIC trainees reported less awareness of the concept of decolonization as well as less negative attitudes toward it. At this time, we are uncertain how best to interpret this finding; we would of course not conclude that this means colonialism was “not so bad.” As part of our broader project, we are conducting follow up interviews with trainees and mentors about challenging situations they have faced and how colonialism may have played a role. We took the opportunity to solicit interviewees feedback on this finding, revealing several possible explanations, such as the more negative views among HIC trainees being motivated by the feelings of guilt seen in our survey; the possibility that cultural differences or power dynamics make LMIC trainees less likely to report negative views; or the fact that decolonizing global health is simply a “hot topic” in global health within HICs. More in-depth exploration of these attitudes is required, and of note, we observed no such difference among LMIC mentors in our limited sample. These findings thus generate additional research hypotheses and motivate additional opportunities for dialogue between and among people from HICs and LMICs alike.

Third, there is a need to account for decolonizing global health throughout global health research training. Few existing curricula explicitly account for these concepts or include them as analytic tools, yet doing so could be fruitful for enhancing global health training. For example, teaching global health trainees about international authorship standards is important by itself [22]; it takes on a new dimension when decolonizing concepts highlight biases of language or ownership of journals in HICs. Similarly, teaching global health trainees about ensuring projects meet local needs is important by itself; it is accentuated in importance when it is understood how global health research funding is dominated by HIC institutions and funders. Global health research trainees are often funded for relative short periods of time (months to a year) and may not be aware of whether or how local needs were taken into account as the project was initiated.

Together, our findings reiterate the importance of reflection upon how we account for decolonizing global health in order to avoid imperialism regarding how global health research training programs are structured [23]. There is an ever present risk that how decolonization is interpreted and implemented will inadvertently perpetuate a colonial mindset by ignoring the voices and decolonizing scholarship of those in LMICs [24].

At the moment, despite a long history of decolonial thinking, incorporating the concept of decolonizing global health into research training is relatively new. Health disparities research frameworks more generally have emphasized the need to detect and understand disparities before reducing them [25]. Seen through this lens, the lack of a common understanding of what decolonizing global health means in research training is problematic; without it, trainees and mentors will be less able to recognize colonial influences in global health research, have trouble understanding their experiences, and lastly be unable to take concrete actions to remediate them. The global health research community needs to intentionally and explicitly create a working definition of what decolonizing global health research training means [26]. This definition should be shared and in common but may not be universal; it should be flexible and even encouraging of dissent and modification over time.

Like all research, our project has limitations. Our study was conducted within a single global health research training program at one point in time. Even though this program includes multiple universities and multiple training sites in different countries, the generalizability of our findings cannot be determined. Our small sample size limited our ability to conduct subanalyses suggested by our data, for example, to evaluate whether attitudes might differ based upon prior global health training experiences, upon type of research being conducted, or perhaps most notably, upon gender or race/ethnicity. We cannot rule out completely a gender effect on responses – and because issues of decolonizing global health may relate to structural bias and disadvantage that can be linked to gender – this area should be explored in future, larger studies. The rapidly evolving nature of dialogues around decolonizing global health also means that understandings of the concept could change rapidly, particularly for trainees, which could affect our results. In addition, given the significant amount of contemporary attention to decolonization, antiracism, and diversity, equity and inclusion generally, it is possible that our findings were affected by social desirability bias (meaning that participants might not have answered truthfully for fear of being judged). Finally, the fact that our survey needed to be developed *de novo* means the items are not completely validated (e.g., in terms of construct validity). Despite these limitations, and given the relative absence of empirical data on this topic, our study provides foundational data and hypotheses to be explored in larger studies.

## Conclusion

The concept of decolonizing global health and its implications for global health research training are as yet not clearly defined. Many are hopeful the attention to decolonization will improve global health equity and empower local communities to improve their own health. Future research should aim toward a common understanding of the concept and should privilege local voices while intentionally leaving room for productive disagreement. Doing so can help ensure efforts to decolonize global health do not inadvertently perpetuate colonial attitudes.

## Data Accessibility Statement

All authors had access to the data and meet ICMJE criteria for authorship.

## Additional files

The additional files for this article can be found as follows:

10.5334/aogh.3961.s1 Supplementary Materials. Table s1 to s3 and Figure s1.

10.5334/aogh.3961.s2 Appendix. Survey Questions.
