# Supplementary material for: Decolonizing Global Health Research: Perspectives from US and International Global Health Trainees
Source: Ann Glob Health. 2023 Feb 6;89(1):9. doi: 10.5334/aogh.3961 (PMC9912860; doi:10.5334/aogh.3961)
Supplement: Supplementary Materials. — Table s1 to s3 and Figure s1. [file agh-89-1-3961-s1.pdf]

**SUPPLEMENTARY MATERIALS/APPENDIX**
DeCamp, Matandika, Chinula, et al. Decolonizing global health research: Perspectives from US and international global health trainees.

**Table S1.** Respondent and non-respondent analysis

| CHARACTERISTIC | Entire program (N=111) | Respondents (n=61) | Non-respondents (n=50 ) | P value |
|---|---|---|---|---|
| Gender¥ | | | | |
| a. Female | 73 (65.8%) | 35 (58.3%) | 38 (74.5%) | 0.073* |
| b. Male | 38 (34.2%) | 25 (41.7%) | 13 (25.5%) | |
| Pre-postdoc (proxy | | | | |
| age?) | 70 (63.1%) | 45 (73.8%) | 25 (50.0%) | 0.010* |
| a. Post | 41 (36.9%) | 16 (26.2%) | 25 (50.0%) | |
| b. Pre | | | | |
| Country | | | | |
| a. HIC | 69 (62.1%) | 36 (59.0%) | 33 (66.0%) | 0.450* |
| b. LMIC | 42 (37.8%) | 25 (41.0%) | 17 (34.0%) | |
| Country | | | | |
| **a. HIC** | **69 (62.1%)** | **36 (50.0%)** | **33 (64.7%)** | |
| HIC & Female¥ | 55 (79.7%) | 27 (75%) | 28 (84.8%) | 0.310* |
| HIC & Male¥ | 14 (20.3%) | 9 (25%) | 5 (15.2%) | |
| **b. LMIC** | **42 (37.8%)** | **24 (40.0%)** | **18 (35.3%)** | |
| LMIC & Female¥ | 18 (42.9%) | 8 (33.3%) | 10 (55.6%) | 0.150* |
| LMIC & Male¥ | 24 (57.1%) | 16 (66.7%) | 8 (44.4%) | |
| Year of Study | | | | |
| a. 2017-2018 | 21 (18.9%) | 11 (18.0%) | 10 (20.0%) | 0.099* |
| b. 2018-2019 | 18 (16.2%) | 8 (13.1%) | 10 (20.0%) | |
| c. 2019-2020 | 27 (24.3%) | 12 (19.7%) | 15 (30.0%) | |
| d. 2020-2021 | 24 (21.6%) | 13 (21.3%) | 11 (22.0%) | |
| e. 2011-2022 | 21 (18.9%) | 17 (27.9%) | 4 (8.0%) | |

** Chi-square test.*

¥ *Analyses by gender do not add up to 61 because one participant did not answer the gender item.*

**Figure S1** Perceptions of the Impact of Colonialism on Various Aspects of Global Health with "Neutral" Category Removed (all respondents)

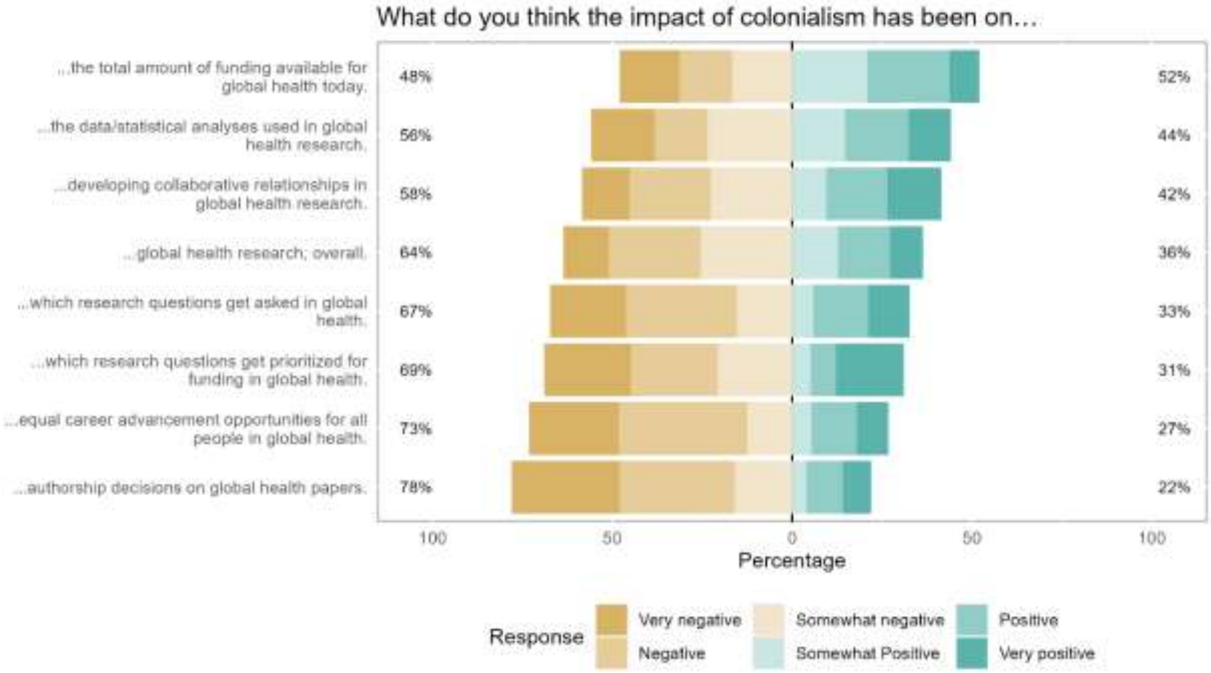

**TABLE S2.** Perceptions of the Impact of Colonialism on Various Aspects of Global Health Among Mentors

| *What do you think the impact of colonialism has been on…* | All respondents (n=26) | HIC respondents (n=16) | LMIC respondents (n=10) | P value |
|---|---|---|---|---|
| **….the total amount of <u>funding</u> available for global health today** | | | | 0.585[ϕ] |
| a. Negative | 12 (46.2%) | 6 (37.5%) | 6 (60.0%) | |
| b. Neutral | 5 (19.2%) | 4 (25.0%) | 1 (10.0%) | |
| c. Positive | 9 (34.6%) | 6 (7.5%) | 3 (30.0%) | |
| **…which <u>research questions</u> get asked in global health.** | | | | 0.680[ϕ] |
| a. Negative | 18 (69.2%) | 10 (62.5%) | 8 (80.0%) | |
| b. Neutral | 2 (7.7%) | 2 (12.5%) | 0 (0.0%) | |
| c. Positive | 6 (23.1%) | 4 (25.0%) | 2 (20.0%) | |
| **…. which <u>research questions get prioritized</u> for funding in global health.** | 18 (69.2%) | 10 (62.5%) | 8 (80.0%) | 0.437[ϕ] |
| a. Negative | 2 (7.7%) | 1 (6.3%) | 1 (10.0%) | |
| b. Neutral | 6 (23.1%) | 5 (31.3%) | 1 (10.0%) | |
| c. Positive | | | | |
| **…developing <u>collaborative relationships</u> in global health research.** | 12 (46.2%) | 6 (37.5%) | 6 (60.0%) | 0.052[ϕ] |
| a. Negative | 7 (26.9%) | 7 (43.8%) | 0 (0.0%) | |
| b. Neutral | 7 (26.9%) | 3 (18.8%) | 4 (40.0%) | |
| c. Positive | | | | |
| **…the <u>data/statistical analyses</u> used in global health research.** | | | | 0.158[ϕ] |
| a. Negative | 12 (46.2%) | 5 (31.6%) | 7 (70.0%) | |
| b. Neutral | 11 (42.3%) | 9 (56.3%) | 2 (20.0%) | |
| c. Positive | 3 (11.5%) | 2 (12.5%) | 1 (10.0%) | |
| **…<u>authorship</u> decisions on global health papers.** | | | | 1.000[ϕ] |
| a. Negative | 19 (73.1%) | 12 (75.0%) | 7 (70.0%) | |
| b. Neutral | 4 (15.4%) | 2 (12.5%) | 2 (20.0%) | |
| c. Positive | 3 (11.5%) | 2 (12.5%) | 1 (10.0%) | |
| **…<u>equal career advancement opportunities</u> for all people in global health.** | | | | 0.549[ϕ] |
| a. Negative | 21 (80.8%) | 13 (81.3%) | 8 (80.0%) | |
| b. Neutral | 4 (15.4%) | 3 (18.6%) | 1 (10.0%) | |

| | | | | |
|---|---|---|---|---|
| c. Positive | 1 (3.9%) | 0 (0.0%) | 1 (10.0%) | |
| **…global health research, overall.**<br><br>a. Negative<br>b. Neutral<br>c. Positive | <br><br>17 (65.4%)<br>4 (15.4%)<br>5 (19.2%) | <br><br>9 (56.3%)<br>3 (18.6%)<br>4 (25.0%) | <br><br>8 (80.0%)<br>1 (10.0%)<br>1 (10.0%) | 0.511[ϕ] |

ϕ *Fisher´s exact test.*

**Table S3.** Perceptions of the Impact of Colonialism on Several Aspects of Global Health (additional analyses)

| *What do you think the impact of colonialism has been on…* | All (n=61) | HIC (n=36) | LMIC (n=25) | P value | Female (n=35) | Male (n=25) | P value | All (mean/ iqr) | HIC (mean/ iqr) | LMIC (mean/ iqr) | P value |
|---|---|---|---|---|---|---|---|---|---|---|---|
| **….the total amount of <u>funding</u> available for global health today**<br>a. Negative<br>b. Neutral<br>c. Positive | <br><br><br>23 (37.7%)<br>13 (21.3%)<br>25 (41.0%) | <br><br><br>12 (33.3%)<br>9 (25.0%)<br>15 (41.7%) | <br><br><br>11 (44.0%)<br>4 (16.0%)<br>10 (40.0%) | 0.602* | <br><br><br>13 (37.1%)<br>7 (20.0%)<br>15 (42.9%) | <br><br><br>9 (36.0%)<br>6 (24.0%)<br>10 (40.0%) | 0.932* | 4.0 / 2.0 | 3.9 / 2.5 | 4 / 3.0 | $0.937^{¥}$<br>$0.908^{⍰}$<br>$0.893^{\Psi}$ |
| **…which <u>research questions</u> get asked in global health.**<br>a. Negative<br>b. Neutral<br>c. Positive | <br><br><br>35 (57.4%)<br>9 (14.8%)<br>17 (27.9%) | <br><br><br>24 (66.7%)<br>7 (19.4%)<br>5 (13.9%) | <br><br><br>11 (44.0%)<br>2 (8.0%)<br>12 (48.0%) | 0.012* | <br><br><br>23 (65.7%)<br>5 (14.3%)<br>7 (20.0%) | <br><br><br>11 (44.0%)<br>4 (16.0%)<br>10 (40.0%) | 0.192* | 3.4 / 3.0 | 2.9 / 2.0 | 4.1 / 4.0 | $0.079^{¥}$<br>$0.028^{⍰}$<br>$0.134^{\Psi}$ |
| **…. which <u>research questions get prioritized</u> for funding in global health.**<br>a. Negative<br>b. Neutral<br>c. Positive | <br><br><br><br>40 (65.6%)<br>3 (4.9%)<br>18 (29.5%) | <br><br><br><br>27 (75.0%)<br>2 (5.6%)<br>7 (19.4%) | <br><br><br><br>13 (52.0%)<br>1 (4.0%)<br>11 (44.0%) | 0.088* | <br><br><br><br>25 (71.4%)<br>3 (8.6%)<br>7 (20.0%) | <br><br><br><br>14 (56.0%)<br>0 (0.0%)<br>11 (44.0%) | 0.065* | 3.4 / 3.0 | 2.3 / 1.5 | 4.0 / 6.0 | $0.219^{¥}$<br>$0.083^{⍰}$<br>$0.113^{\Psi}$ |
| **…developing <u>collaborative relationships</u> in global health research.**<br>a. Negative<br>b. Neutral<br>c. Positive | <br><br><br><br><br>31 (50.8%)<br>8 (13.1%)<br>22 (36.1%) | <br><br><br><br><br>25 (69.4%)<br>5 (13.9%)<br>6 (16.7%) | <br><br><br><br><br>6 (24.0%)<br>3 (12.0%)<br>16 (64.0%) | $<0.001^{\phi}$ | <br><br><br><br><br>20 (57.1%)<br>6 (17.1%)<br>9 (25.1%) | <br><br><br><br><br>10 (40.0%)<br>2 (8.0%)<br>13 (52.0%) | $0.120^{\phi}$ | 3.8 / 4.0 | 3.1 / 2.0 | 4.9 / 2.0 | $<0.001^{¥}$<br>$<0.001^{⍰}$<br>$<0.001^{\Psi}$ |
| **…the <u>data/statistical analyses</u> used in global health** | | | | <0.001* | | | 0.568* | 3.9 / | 3.6 / | 4.3 / | $0.083^{¥}$ |

| | | | | | | | | | | | |
|---|---|---|---|---|---|---|---|---|---|---|---|
| **research.**<br>a. Negative<br>b. Neutral<br>c. Positive | 19 (31.2%)<br>27 (44.3%)<br>15 (24.6%) | 11 (30.6%)<br>22 (61.1%)<br>3 (8.3%) | 8 (32.0%)<br>5 (20.0%)<br>12 (48.0%) | | 12 (34.3%)<br>16 (45.7%)<br>7 (20.0%) | 7 (28.0%)<br>10 (40.0%)<br>8 (32.0%) | | 1.0 | 1.0 | 3.0 | 0.100▯<br>0.001 Ψ |
| **…authorship decisions on global health papers.**<br>a. Negative<br>b. Neutral<br>c. Positive | 39 (63.9%)<br>11 (18.0%)<br>11 (18.0%) | 27 (75.0%)<br>5 (13.9%)<br>4 (11.1%) | 12 (48.0%)<br>6 (24.0%)<br>7 (28.0%) | 0.092ϕ | 24 (68.6%)<br>5 (14.3%)<br>6 (17.1%) | 14 (56.0%)<br>6 (24.0%)<br>5 (20.0%) | 0.570ϕ | 3.0 /<br>2.0 | 2.7 /<br>1.5 | 3.4 /<br>4.0 | 0.217¥<br>0.121 ▯<br>0.251 Ψ |
| **…equal career advancement opportunities for all people in global health.**<br>a. Negative<br>b. Neutral<br>c. Positive | 41 (67.2%)<br>5 (8.2%)<br>15 (24.6%) | 28 (77.8%)<br>3 (8.3%)<br>5 (13.9%) | 13 (52.0%)<br>2 (8.0%)<br>10 (40.0%) | 0.060ϕ | 25 (71.4%)<br>4 (11.41%)<br>6 (17.1%) | 15 (60.0%)<br>1 (4.0%)<br>9 (36.0%) | 0.257ϕ | 3.0 /<br>2.0 | 2.6 /<br>1.5 | 3.7 /<br>4.0 | 0.094¥<br>0.043▯<br>0.202 Ψ |
| **…global health research, overall.**<br>a. Negative<br>b. Neutral<br>c. Positive | 35 (57.4%)<br>6 (9.8%)<br>20 (32.8%) | 24 (66.7%)<br>4 (11.1%)<br>8 (22.2%) | 11 (44.0%)<br>2 (8.0%)<br>12 (48.0%) | 0.129ϕ | 21 (60.0%)<br>4 (11.4%)<br>10 (28.6%) | 13 (52.0%)<br>2 (8.0%)<br>10 (40.0%) | 0.682ϕ | 3.6 /<br>3.0 | 3.2 /<br>2.0 | 4.2 /<br>3.0 | 0.059¥<br>0.045▯<br>0.134 Ψ |

** Chi-square test.*

ϕ *Fisher´s exact test.*

¥ *Mann Whitney test.*

▯ *T-test.*

Ψ *Median-test.*
