# Supplementary material for: Decolonizing Global Health Research: Perspectives from US and International Global Health Trainees
Source: Ann Glob Health. 2023 Feb 6;89(1):9. doi: 10.5334/aogh.3961 (PMC9912860; doi:10.5334/aogh.3961)
Supplement: Appendix. — Survey Questions. [file agh-89-1-3961-s2.pdf]

INTRODUCTION

The purpose of this project is to better understand the experiences of trainees and mentors in the UJMT program. We will use what we learn to develop new educational materials on global bioethics for the UJMT. There is no medical risk to your participation. Your answers to these questions will be confidential. We will not share your answers with your mentors or the UJMT program in identifiable form. Please, be as honest as you can – this will help us prepare training materials as needed. Participation is voluntary.

1. ***If you complete the entire survey, you will be entered into a drawing to receive one of 3 new iPads. Do you want to be entered into this drawing?**
    a. Yes
    b. No

[NEW PAGE]

DEMOGRAPHIC DATA

Before we get started, we want to learn a little bit about you and your experience with UJMT.

2. ***What was your UJMT Training year?**
    a. 2021-2022 (current year)
    b. 2020-2021
    c. 2019-2020
    d. 2018-2019
    e. 2017-2018

    ***IF a or b.: You indicated above that you were a UJMT trainee during the COVID-19 pandemic. We want to know if COVID-19 affected your training.**

    2a. Which of the following describes your time with UJMT during COVID-19?

    a. COVID-19 prevented me from traveling to my research site.
    b. COVID-19 affected my travel – I was delayed, or went less often or for less time, to my research site.
    c. COVID-19 did not affect my travel – I traveled normally to my research site.

    2b. Please rate your agreement with the following statements.

	**Strongly Disagree**	**Disagree**	**Neutral**	**Agree**	**Strongly Agree**
My research topic significantly changed because of COVID.					

It was more difficult to connect with mentors in the program because of COVID-19.					
It was more difficult to connect with other trainees in the program because of COVID-19.					

2c. IF a or b.: Could you please share anything else with us about how COVID-19 affected your work in UJMT?

[OPEN TEXT BOX] or Skip

[NEW PAGE]

3. ***Which of the following best describes your experience in the UJMT Program?**
   a. I went from a high income country, such as the US, to a low- or middle-income country.
   b. I went from a low- or middle-income country to a high income country.
   c. My training experience occurred within my own country.
   d. I went from one low- or middle-income country to another low- or middle-income country.

4. ***Are you a US Citizen?**
   a. Yes
   b. No

   4a. If YES -> Do you consider yourself a member of an underrepresented population in medicine or science? [In the United States, “underrepresented” refers to Blacks or African Americans, Hispanics or Latinos, American Indians or Alaska Natives, or Native Hawaiians and other Pacific Islanders.]

   a. Yes
   b. No

5. **At the time you were a UJMT Trainee, which of the following best describes your background?** *Choose all that apply:*
   a. I was pursuing my MD degree
   b. I was pursuing my PhD degree
   c. I already had my MD
   d. I already had my PhD
   e. Other: _______________

5a. If b **or** d → **Which of the following best describes the PhD you have or are pursuing?**

a. Basic Science
b. Public Health
c. Social Science
d. Other:________________

6. ***Which of the following best describes the research you carried out as part of the UJMT program?** *Choose all that apply:*
    a. Basic science
    b. Secondary data analysis
    c. Clinical trial
    d. Public health or health education
    e. Implementation science
    f. Other: __________________

[NEW PAGE]

For the purposes of this survey:

We define global health training <u>broadly</u>. For US-based students, examples might be short-term trips to another country for the sake of health education, research or service (e.g., public health or health care delivery). For international students, examples might include short-term trips to other countries as well as training experiences within one's own country to unfamiliar areas.

We also define global bioethics <u>broadly</u> to include research ethics, cultural sensitivity, and other ethics issues that come up in global health research and training settings.

CLICK TO CONTINUE

[NEW PAGE]

7. ***How many prior experiences did you have (before your year with UJMT) where you were immersed in a global health training program, at home or abroad, broadly defined?**
    a. Never.
    b. 1-2 times
    c. 3-5 times
    d. More than 5 times

8. **Did you have prior experience or training in bioethics *in general* (i.e., not necessarily "global") before the start of your trainee year?**
    a. Yes
    b. No

    8a. If YES -> Tell us about your experience or training in bioethics (*Check all that apply):*

    a. I have read about bioethics on my own
    b. I have taken online modules (for example, CITI training)
    c. I have taken at least one undergraduate-level course on bioethics
    d. I have received master's level training in bioethics
    e. I have served on a research ethics committee or institutional review board
    f. Other _________________

[NEW PAGE]

9. ***At the time you began your year with UJMT, did you have prior experience or training in specifically global bioethics?**
    a. Yes
    b. No

    9a. If YES -> Tell us about your experience or training in global bioethics (*Check all that apply):*
    a. I have read about global bioethics on my own
    b. One of my bioethics courses included a section on global health
    c. I have taken a course on global bioethics
    d. I have master's level training in global bioethics
    e. I have PhD-level training in global bioethics
    f. Other_______________________

    9b. If YES -> When you think about this prior global bioethics training, how helpful was it managing the specific bioethics challenges you experienced or witnessed in the UJMT program?
    a. Not at all
    b. A little helpful
    c. Somewhat helpful
    d. Very helpful

[NEW PAGE]

10. **When you started your training time with the UJMT program, how prepared were you to:**

	Not at all prepared	Somewhat prepared	Very prepared	Completely prepared
**Identify a global bioethics issue**				
**Bring a global bioethics issue up to your mentor**				
**Resolve a global bioethics issue on your own**				

11. **Right now, how confident are you in your ability to:**

	Not at all confident	Somewhat confident	Very confident	Completely confident
**Identify a global bioethics issue**				
**Bring a global bioethics issue up to your mentor**				
**Resolve a global bioethics issue on your own**				

12. ***Please rate your overall agreement with the following statement:**
    More training in global bioethics is needed to better achieve the goals of the UJMT program.
    a. Strongly disagree
    b. Disagree
    c. Neutral
    d. Agree
    e. Strongly agree

BASELINE KNOWLEDGE/ATTITUDES

Now we would like to ask a few questions about a concept that has been getting attention lately: decolonizing global health.

13. ***Please rate your overall knowledge of the concept of "decolonization" of global health.**
    a. I have never heard of this.
    b. I know a little bit.
    c. I know a lot
    d. I'm an expert.

[NEW PAGE]

14. ***Tell us, in your own words and in 1 sentence, what the phrase "decolonizing global health" means to you. It is OK to say, "I don't know."**

[OPEN TEXT BOX]

15. **In the space below, can you give an example of something you experienced – good or bad - within UJMT that related to the concept of "decolonization of global health"?** (*REMEMBER:* We will keep your answers to this survey confidential and will not share them with your mentors or the UJMT program in identifiable form.)

[OPEN TEXT BOX] or Explicit Skip

[NEW PAGE]

16. **Please rate your overall agreement with the following statements about decolonizing global health.** (5-point Likert scale, Strongly Disagree to Strongly Agree)

    a. I am *supportive* of the current movement toward decolonization of global health.
    b. I am *worried* that the current movement toward decolonization will do more harm than good.
    c. I feel "*excited*," because decolonization will give more opportunities for people in low- or middle-income countries to succeed in global health research.
    d. I feel "*defensive*," like I am being accused of doing something wrong.
    e. I feel "*hopeful*" that decolonization will actually reduce global health inequalities.
    f. I feel "*guilty*," because I personally benefitted from colonialism.

17. ***In your opinion, how much does the history of colonization affect the way bioethical issues present in the UJMT program?** (5 point slider bar + "I don't know")
    a. No effect at all
    b.
    c.
    d.
    e. A very significant effect
    f. I don't know

[NEW PAGE]

18. ***Colonialism can be defined as when one group of people conquers or rules over another, as has happened throughout human history. Some people believe colonialism has the potential to impact events today.** [Seven point slider bar or 7-point Likert scale.]

What do you think the impact of colonialism has been on…	**-3 Very Negative**	**-2**	**-1**	**0**	**1**	**2**	**3 Very Positive**
…the total amount of funding available for global health today.							

…which research questions get asked in global health.							
…which research questions get prioritized for funding in global health.							
…developing collaborative relationships in global health research.							
…the data/statistical analyses used in global health research.							
…authorship decisions on global health papers.							
…equal career advancement opportunities for all people in global health.							
…global health research, overall.							

[NEW PAGE]

ASSESSMENT OF BIOETHICAL ISSUES

For the next few questions, we want to learn what you think are the most important issues to include in NEW global bioethics training materials we are going to develop for UJMT.

19. ***Imagine you were building a <u>NEW</u> training manual for bioethics for the UJMT training program. If you had to pick one global bioethics issue to include, what would it be?**

    [OPEN TEXT BOX]

[NEW PAGE]

20. ***Below are five areas where individuals sometimes experience or witness challenges as part of a global health training program related to RESEARCH ETHICS.**

    Please order them from 1-5 (1= most important to include; 5= least important to include), in terms of how important they would be to include in a <u>new</u> bioethics training manual.

    - ☐ Informed Consent
    - ☐ Data privacy and confidentiality
    - ☐ Data ownership and data custodianship (i.e., who owns data and samples, or where they are analyzed)
    - ☐ Authorship
    - ☐ Ensuring the research project meets local needs

21. ***Is there another issue you think should be on this list?**
   - **a.** Yes
   - **b.** No

   20a. If YES -> Please describe this issue: [OPEN TEXT BOX]

   20b. How would you rank this issue on a scale of 1-5 (1= most important to include; 5= least important to include)? 1 2 3 4 5

22. ***In question 20, you rated [INSERT ISSUE FROM #20] as most important to include. What role do you think the impact of colonialism plays in contributing to that issue?**
   - **a.** No role
   - **b.** Very little role
   - **c.** Somewhat of a role
   - **d.** A major role
   - **e.** I don't know

   22a. Could you please share in the box below why you answered the question this way.

   [OPEN TEXT BOX] or Explicit Skip

23. *** Below are five areas where individuals sometimes experience or witness challenges as part of a global health training program related to the OVERALL PROGRAM.**

   Please order them from 1-5 (1= most important to include; 5= least important to include), in terms of how important they would be to include in a <u>new</u> bioethics training manual.

   - ☐ Managing limited resources
   - ☐ Sustainability
   - ☐ Dealing with real or perceived power imbalances
   - ☐ Unintentional burdens on the local community
   - ☐ Differences in cultural norms

24. ***Is there another issue you think should be on this list?**
   - **a.** Yes
   - **b.** No

   22a. If YES -> Please describe this issue: [OPEN TEXT BOX]

22b. How would you rank this issue on a scale of 1-5 (1= most important; 5= least important)? 1 2 3 4 5

25. **In questions 23, you rated [INSERT ISSUE FROM QUESTION 23] as most important to include. What role do you think the impact of colonialism plays in contributing to that issue?**
    a. No role
    b. Very little role
    c. Somewhat of a role
    d. A major role
    e. I don't know

24a. Could you please share in the box below why you answered the question this way.

[OPEN TEXT BOX] or Explicit Skip

26. *** Below are five areas where individuals sometimes experience or witness challenges as part of a global health training program related to THE TRAINEE EXPERIENCE.**

    Please order them from 1-5 (1= most important to include; 5= least important to include), in terms of how important they would be to include in a <u>new</u> bioethics training manual.

    - ☐ Performing duties or tasks outside my scope
    - ☐ Moral distress (knowing the right thing to do, but being unable to do it)
    - ☐ Preferential treatment of some trainees over others
    - ☐ Not getting credit for what I had done
    - ☐ Benefitting at others' expense

27. ***Is there another issue you think should be on this list?**
    a. Yes
    b. No

26a. If YES -> Please describe this issue: [OPEN TEXT BOX]

25b. How would you rank this issue on a scale of 1-5? (1= most important; 5= least important) 1 2 3 4 5

28. *** In questions 26, you rated [INSERT ISSUE] as most important to include. What role do you think the impact of colonialism plays in contributing to that issue?**
    a. No role
    b. Very little role
    c. Somewhat of a role
    d. A major role
    e. I don't know

27a. Could you please share in the box below why you answered the question this way.

[OPEN TEXT BOX] or Explicit Skip

NEEDS ASSESSMENT

[NEW PAGE]

Now we'd like to learn more about how you think we could improve bioethics education on these topics in UJMT.

29. **Do you have examples of bioethics training materials you have liked in the past? If so, please describe them:**

[OPEN TEXT BOX] or Explicit Skip

[NEW PAGE]

30. ***Please rank the following methods of teaching global bioethics in order from 1 (I would like this the most) to 7 (I would like this the least).**
    - ☐ An online webinar
    - ☐ A list of readings
    - ☐ A prepared case & analysis that I can read on my own
    - ☐ A scripted role play involving my peers and/or mentors
    - ☐ A case study that I read and analyze with my mentor
    - ☐ A case study that I read and analyze with a <u>single peer</u> or colleague
    - ☐ A case study that I read and analyze with a <u>small group</u> of my peers or colleagues

31. ***If you were to receive new materials to learn about global bioethics during your 1-year training program in UJMT, which of the following describes how much time you'd be able to spend on it?**
    - **a.** 90-120 minutes every 3 months (6-8 hours a year)
    - **b.** An hour every month (12 hours a year)
    - **c.** An hour every other month (6 hours per year)
    - **d.** I don't have time for this at all
    - **e.** Other Suggestion: _____________

32. **If you were to receive new materials to learn about global bioethics during your 1-year training program with UJMT, when would be the best time to receive this additional training?**
    - a. Before the program-wide orientation in the summer

b. During the program-wide orientation in the summer
c. Within a month or two of starting the program
d. Halfway through the training year
e. Near the end of the program
f. At regular points throughout the program year
g. Other: ___________________

33. ***Which of the following do you think would help MOST in managing global bioethics issues that could come up during the UJMT program?** *(Please choose up to 3.)*
    a. Additional formal instruction in global health ethics
    b. A list of readings or books
    c. Connecting with a trainee who had been at my site before
    d. More opportunities to discuss ethical issues in real-time (e.g., informal webinars during the year)
    e. Regularly scheduled "bioethics meeting" times that could be used to talk about case studies or real situations with other UJMT <u>peers or trainees</u>
    f. Regularly scheduled "bioethics meeting" times that could be used to talk about case studies or real situations with UJMT <u>mentors</u>
    g. A book or journal club that meets every 2-3 months
    h. Dedicated sessions with my mentor where I can bring up ethical issues
    i. Other ideas: ________________________

[NEW PAGE]

MENTOR/MENTEE RELATIONSHIP

34. ***If a bioethical issue came up during your time in the UJMT, who would you be <u>most</u> <u>likely</u> to discuss it with?**
    a. My mentor at my home institution
    b. My mentor at my research/training site
    c. Another trainee in the program
    d. Other: _________________________

35. **If something you thought was a bioethical issue came up during your time in the UJMT, how would you (or did you) try to address it?**

[OPEN TEXT BOX] or Skip

[NEW PAGE]

CLOSING

36. Would you be willing to talk with us more about any of your experiences with global bioethics and/or the concept of decolonizing global health? If so, we may contact you for a confidential interview by phone or video conferencing. You will be reimbursed for your time.

a. Yes → Please provide the best way to reach you (email or phone): ______________________

b. No

Thank you for taking this survey. If you indicated you would like to be considered for the iPad drawing, please confirm your best contact email address here: ___________________________
